# Exercise-Mediated Neurogenesis in the Hippocampus via BDNF

**DOI:** 10.3389/fnins.2018.00052

**Published:** 2018-02-07

**Authors:** Patrick Z. Liu, Robin Nusslock

**Affiliations:** Department of Psychology, Northwestern University, Evanston, IL, United States

**Keywords:** neurotrophin, TrkB, subgranular zone, neuroprotective, dentate gyrus

## Abstract

Exercise is known to have numerous neuroprotective and cognitive benefits, especially pertaining to memory and learning related processes. One potential link connecting them is exercise-mediated hippocampal neurogenesis, in which new neurons are generated and incorporated into hippocampal circuits. The present review synthesizes the extant literature detailing the relationship between exercise and hippocampal neurogenesis, and identifies a key molecule mediating this process, brain-derived neurotrophic factor (BDNF). As a member of the neurotrophin family, BDNF regulates many of the processes within neurogenesis, such as differentiation and survival. Although much more is known about the direct role that exercise and BDNF have on hippocampal neurogenesis in rodents, their corresponding cognitive benefits in humans will also be discussed. Specifically, what is known about exercise-mediated hippocampal neurogenesis will be presented as it relates to BDNF to highlight the critical role that it plays. Due to the inaccessibility of the human brain, much less is known about the role BDNF plays in human hippocampal neurogenesis. Limitations and future areas of research with regards to human neurogenesis will thus be discussed, including indirect measures of neurogenesis and single nucleotide polymorphisms within the BDNF gene.

## Introduction

For centuries, researchers have sought to elucidate the mechanisms behind the axiom that a healthy body leads to a healthy mind. It has now been established that exercise, even among minimal commitment exercise routines, has an array of robust effects on the brain, such as enhanced memory, mood, cognitive functioning, plasticity, and learning capabilities (Erickson et al., 2011; Spalding et al., 2013; Phillips et al., 2014). Most notably, exercise has been implicated in having anti-depressant effects and counteracting disease or age-related mental impairment and atrophy, such as Alzheimer's disease or dementia (Laurin et al., 2001). Yet, until recently, the intermediaries between exercise and its health benefits have not been well-understood.

However, it has been shown that—contrary to the age-old notion that the number of neurons in the brain remains static after prenatal and neonatal development—new neurons can be generated in the adult brain via a process known as neurogenesis, which can attenuate the deleterious effects of neurodegeneration (van Praag et al., 1999). This phenomenon has been linked to exercise, with a significant portion of subsequent neural growth occurring in the dentate gyrus of the hippocampus (Cotman and Berchtold, 2002). Since the hippocampus is critical for memory consolidation and learning, the generation of new neurons and increased plasticity in this brain region may explain the improved cognition and emotional state that accompanies exercise (Gandy et al., 2017; Trinchero et al., 2017). Furthermore, preliminary research has suggested that neurogenesis may also occur in numerous other areas of the brain, including the amygdala and hypothalamus, which may explain the diversity of exercise-derived benefits (Fowler et al., 2008). However, this research is not as extensive or conclusive as hippocampal neurogenesis research, nor is the extent to which neurogenesis occurs in other brain regions as robust as it is in the hippocampus, with the exception of the olfactory bulb (Cotman et al., 2007).

As such, this review will exclusively summarize the effects of exercise on neurogenesis in the hippocampus. This paper will also explicate a key molecule, brain-derived neurotrophic factor (BDNF), that has been shown to modulate neurogenesis and how exercise influences BDNF levels. Lastly, due to discrepancies between rodent and human studies, limitations and future areas of investigation with regards to neurogenesis in humans will be discussed.

## Exercise and hippocampal neurogenesis

In rodents, hippocampal neurogenesis as a function of exercise has been extensively demonstrated and replicated. To test this, rodents are injected with bromodeoxyuridine (BrdU), which signify actively mitotic cells and are incorporated by daughter cells, thereby allowing the tracing of cell division (del Rio and Soriano, 1989). In some of the earliest work in this field, it was shown that mice allowed to voluntarily exercise on a running wheel exhibited enhanced neurogenesis in the dentate gyrus. By utilizing BrdU as a tracing molecule, it was observed that exercise not only increased proliferation of the progenitor cells in the subgranular zone, but also increased their survival rate as they differentiated and matured (van Praag et al., 1999; Seri et al., 2001; for reviews of neural progenitors and lineage progression see Weissman et al., 2001; Seri et al., 2004; Göritz and Frisén, 2012).

Although it is much more difficult to study exercise-mediated neurogenesis in humans, there is significant evidence that neurogenesis occurs in the adult human brain, especially in the dentate gyrus. Indeed, exercise has been shown to increase the size of the hippocampus in human adults (Erickson et al., 2011). Through postmortem tissue analysis of cancer patients administered BrdU, it has been shown that mature granule neurons are continually generated from the subgranular zone, even in the later stages of life (Eriksson et al., 1998). Interestingly, the participants in this study were not assigned to exercise conditions, and since they were cancer patients near death, it is unlikely they participated in any exercise regimen. This suggests that the hippocampus has the capability to generate new neurons in adulthood independent of exercise. Later sections in this review, however, provide evidence that exercise accentuates neurogenesis in humans and addresses how the amount of exercise modulates the degree of neurogenesis.

## BDNF mediation of hippocampal neurogenesis

Given these early findings establishing a connection between exercise and hippocampal neurogenesis, researchers next turned to elucidating the biological underpinnings. One of the strongest candidates for bridging the gap between exercise and neurogenesis is BDNF, a growth factor categorized under the neurotrophin family widely expressed in the brain and throughout the rest of the central nervous system (Salehi et al., 2003). Early research on this molecule found that during development in mice, BDNF expression is low during prenatal development, but then increases during the first few weeks after being born and peaks during the shift from embryonic to adult neurogenesis (Bath et al., 2012). This provides key insight into its potential for facilitating neurogenesis, which then spurred much more research interest in its connection to neurogenesis.

### BDNF

As a whole, the neurotrophin family polypeptides are vital to the regulation of the neural processes in neurogenesis, such as proliferation, differentiation, maturation, and plasticity. Within this family, BDNF exhibits the highest degree of expression in the brain, and is primarily synthesized there during exercise (Reichardt, 2006). BDNF can also enter the brain via freely diffusing across the blood brain barrier (Pan et al., 1998; Mousavi and Jasmin, 2006). Furthermore, during exercise, proteins and their metabolic derivatives secreted from peripheral muscles, such as cathepsin B and FNDC5/irisin, also cross the blood brain barrier to mediate BDNF expression in the hippocampus and subsequent neurogenesis and memory improvement (Wrann et al., 2013; Moon et al., 2016). Indeed, mice injected with skeletal muscle endurance factors had elevated levels of hippocampal neurogenesis and increased spatial memory (Kobilo et al., 2010).

BDNF functions by binding to tropomyosin receptor kinase B (TrkB), which is largely expressed in hippocampal neurons. Upon binding, the BDNF-TrkB complex is then internalized into the neuron and serves as a docking site for numerous signaling cascades, protein phosphorylation cascades, and secondary signaling systems (Huang and Reichardt, 2003; Nykjaer et al., 2005; Yoshii and Constantine-Paton, 2010; for review of signaling pathways see Phillips et al., 2014). Through these pathways, BDNF can exert significant regulatory control over many facets of a neuron's function and thereby influence how these neurons function as a whole within the hippocampus.

### BDNF and TrkB influences on the hippocampus

Given the vast array of possible signaling pathways through which BDNF can operate, it is not surprising that BDNF expression impacts multiple aspects of the hippocampus, including cognitive functioning and neurogenesis. In terms of cognitive processes, higher levels of BDNF expression have been implicated in long-term potentiation of neurons and synaptic plasticity, which may explain why higher levels of BDNF expression are associated with enhanced spatial and verbal memory and recognition capabilities, and may also counteract the effects of chronic stress and cognitive decline (Korte et al., 1995; Voss et al., 2013b; Wang et al., 2015; Liu and Nusslock, 2018; for reviews of plasticity see Lu et al., 2013, 2014; Karpova, 2014; Leal et al., 2015). Indeed, in mice, BDNF is required for hippocampal neurogenesis spurred by an enriched environment, and BDNF has been shown to have coordinated effects with antidepressants on neuronal progenitor cells in the hippocampus (Sairanen et al., 2005; Rossi et al., 2006). Furthermore, decreased BDNF levels, especially in older adults, may lead to compromised memory, neurodegeneration, and various other cognitive impairments found in Alzheimer's disease (for review, see Zuccato and Cattaneo, 2009).

To study BDNF's role in neurogenesis, researchers have utilized BDNF and TrkB knockdown mice and also exogenous factors to overexpress BDNF. In BDNF knockdown mice, it was observed that the neural stem cells in the subgranular zone proliferated significantly less than in wild type mice. Moreover, significantly more of the stem cells that did proliferate died in the BDNF knockdown mice before fully differentiating and maturing (Lee et al., 2002). These results suggest that at the very least, BDNF is necessary for a basal level of neurogenesis. Further work corroborated these findings by demonstrating that in TrkB knockdown mice, both proliferation and differentiation of neural stem cells are significantly reduced, thereby suggesting that BDNF and TrkB are functionally dependent upon each other and that altering TrkB responsivity affects neurogenesis equally as altering BDNF expression levels (Bartkowska et al., 2007; Li et al., 2008). Removal of TrkB also stunted dendritic growth and adult progenitor integration into the hippocampus in adult mice, which led to more pronounced anxious behavior (Bergami et al., 2008).

However, when BDNF expression levels were exogenously increased in mice with wild type TrkB, these effects were reversed and even accentuated. Even when administered a single injection of BDNF, substantially more neurons were recruited from neuronal progenitor cells (Benraiss et al., 2001). And when chronically stimulated with BDNF expression, a significant increase in net neurogenesis was observed in the dentate gyrus (Quesseveur et al., 2013). In fact, many individual studies have found that increased BDNF expression robustly promotes the *in vivo* proliferation, triggering of differentiation, axonal path migration, and maturation of the neural stem cells in the dentate gyrus (Korte et al., 1995; Reichardt, 2006; Nakata and Nakamura, 2007; Waterhouse et al., 2012). Through an autocrine loop initiated by BDNF, these newly generated neurons are also better protected against cell death (Acheson et al., 1995).

Therefore, considering both the knockdown and overexpression studies in tandem, there is an established connection between BDNF and neurogenesis. Since BDNF also promotes neuronal survival and enhanced nerve transmission via long-term potentiation, this combination of neurogenesis and optimized neuronal functioning significantly improves cognitive performance and protects against neurodegenerative phenomena.

### Exercise and BDNF expression

Thus far, both exercise and BDNF have been shown to be associated with increased neurogenesis. Further research has extended this to show that treadmill exercise in mice and aerobic exercise in humans increases BDNF expression by regulating BDNF gene expression in the hippocampus (Kim et al., 2015). This process is largely mediated by neurotransmitter and neuroendocrine systems, with extensive literature supporting acetylcholine (ACh) as a key regulator (Knipper et al., 1994).

In mice allowed to voluntarily engage in wheel running, an increase in BDNF mRNA levels in the dentate gyrus was observed after only a few days of exercise (Neeper et al., 1995). Surprisingly, these levels were maintained throughout several weeks of exercise and corresponded to proportional increases in BDNF protein expression (Russo-Neustadt et al., 1999). When the exercise conditions were supplemented with antibodies blocking TrkB, however, the mice had attenuated learning capabilities involving the hippocampus. Furthermore, these mice also lacked synaptic-specific proteins in the hippocampus, thereby demonstrating that BDNF signaling is necessary to allow the benefits of exercise to manifest (Vaynman et al., 2004, 2006).

Research has also shown that metrics of overall health quality in humans follow a dose-dependent relationship with the duration and intensity of exercise, with the best outcomes linked to moderate exercise (Larson et al., 2006). Further work illustrates that mice show greater improvements in acquisition and retention based learning in hippocampus-dependent tasks following long-term exercise rather than shorter regimes of exercise (Handschin and Spiegelman, 2008; Parachikova et al., 2008; Ploeger et al., 2009). It was found that in mice, even just one session of exercise increased BDNF levels. This effect, however, became amplified following a period of exercise in mice that regularly exercised, with an increased response in BDNF levels relative to mice after just a single session of exercise (Johnson et al., 2003; van Praag et al., 2005; Rasmussen et al., 2009). Consistent with these findings is a meta-analysis of 29 studies spanning 1,111 human participants that analyzed BDNF expression levels across various exercise paradigms. However, many of the studies only examined moderate exercise, and several studies did not report intensity level. Interestingly, considerable evidence from this meta-analysis suggests that humans also experience a dose-response relationship in which each session of exercise corresponds to a dose of increased BDNF expression. Furthermore, regular exercise in moderate amounts has been shown to increase the magnitude of BDNF expression following individual sessions of exercise (Szuhany et al., 2015).

There is not a perfect positive correlation, however, between the amount and intensity of exercise and BDNF expression levels and subsequent health benefits. Extreme exercise has been shown to disrupt a number of metabolic and physiological processes and lead to impaired cognitive performance in humans (Aguiló et al., 2005). Since oxygen is rapidly metabolized during physical exertion, reactive oxygen species (ROS) are naturally produced as a metabolic byproduct. When produced at high levels, such as during bouts of intense exercise, ROS can lead to oxidative damage and increased cellular mortality in both rodents and humans (Radak et al., 2016). Moderate levels of exercise enforce the human body's antioxidant defense system, but extreme levels of exercise lead to the generation of more ROS than the antioxidant system can defend against, thereby allowing their accumulation as oxidative stress (Mastaloudis et al., 2001). In fact, when treated with hydrogen peroxide, a potent ROS, hippocampal cell cultures taken from rodents showed an inverse relationship between BDNF expression levels and hydrogen peroxide concentration (Kwon et al., 2013). The *in vivo* production of BDNF as a function of ROS production, however, is less clear and warrants further study.

## Limitations and future directions

Both optimized health outcomes and increased BDNF levels have been shown to follow moderate exercise in humans. Given how these results are consistent in mice studies and how BDNF is a highly conserved growth factor across numerous species, it is likely that BDNF also heavily mediates exercise-induced neurogenesis in the dentate gyrus in humans. However, due to ethical reasons, it is difficult to conclusively test this since brain samples cannot be readily collected from human participants following various exercise paradigms.

Furthermore, even if neurogenesis could be tested in humans following different exercise routines, it would be nearly impossible to track the time course of new neuronal development since this would require harvesting brain tissue postmortem. As such, neurogenesis studies in humans need to rely on indirect measures, such as administering cognitive tests related to memory consolidation or measuring neurobiological indicators of neurogenesis and plasticity, such as BDNF (Voss et al., 2013a). A potential indirect measurement that has been established is the correlation between cerebral blood volume in the dentate gyrus, cognitive enhancements, and adult neurogenesis as a function of exercise (Pereira et al., 2007). Although this correlation is robustly established in mice, it is still difficult to conclusively connect cerebral blood volume to neurogenesis in humans. Accompanying studies in rodents should therefore definitively establish how indicative these indirect measures are of neurogenesis and also seek to determine causality so that human neurogenesis can be better assessed and analyzed following exercise or BDNF supplementation. Although discrepancies between animal model and human studies exist (Spalding et al., 2013; Paredes et al., 2016), the outlook for future studies should be optimistic since a combined approach of analyzing BDNF levels, cognitive functioning, and cerebral blood volume could provide significant evidence suggesting adult neurogenesis.

Another factor to consider in this field of research is the contribution of gene-environment interactions. The most heavily studied single nucleotide polymorphism (SNP) in the BDNF gene is rs6265, otherwise known as val66met. This SNP reduces BDNF's distribution within neurons and also its protein binding affinity, which has been linked to neuropathology and cognitive deficiencies (Chiaruttini et al., 2009). In a meta-analysis of 399 healthy participants, met carriers were found to have significantly decreased hippocampal volumes (Hajek et al., 2012). In an extensive longitudinal study, the presence of rs6265 robustly predicted the gradual decline of hippocampal volume and also subsequent skilled task performance (Sanchez et al., 2011). This SNP has also been associated with the occurrence of major depressive disorder, anxiety disorders, suicidal tendencies, and depressive episodes in bipolar disorder and in patients with Alzheimer's disease (Kim et al., 2008; Terracciano et al., 2010).

Interestingly, there appears to be a bidirectional relationship between various BDNF polymorphisms and the environment that future studies should account for. The presence of a val2met substitution has been shown to hinder working memory and other aspects of cognition, yet high levels of exercise counteracted these cognitive impairments, suggesting that exercise may be able to modulate genetic influences (Erickson et al., 2013). However, in other forms of BDNF polymorphisms, adenine alleles in the polymorphisms have been shown to decrease compliance in participants to engage in highly demanding exercise regimens (Bryan et al., 2013). As such, the interaction of BDNF polymorphisms with exercise is difficult to disambiguate and needs further elucidation.

Lastly, another area for further study will be to directly measure BDNF expression as a function of exercise intensity, duration, and sedentary status. Although many studies mentioned in this review analyzed BDNF expression from moderate exercise, much less is known about how different levels of exercise intensity directly affect BDNF levels and subsequent neurogenesis. Additionally, in the human studies, a person's sedentary status described as sedentary, regular exercise, or athlete was either not noted or controlled for via randomization studies. As such, it is also unclear how sedentary status combined with exercise intensity can modulate BDNF expression (Szuhany et al., 2015).

Despite what is still yet unknown regarding exercise-mediated neurogenesis and BDNF, it is nonetheless known that exercise has immense benefits for physical, psychological, developmental, and social factors. Exercise, when pursued in moderation, not only serves as a robust method for improving physical health, but also serves as a preventative and protective measure against numerous neurological and mental diseases. Neurogenesis is still a poorly understood topic, and as the role and benefits of exercise are continually being better elucidated, future research initiatives on exercise will undoubtedly uncover more benefits on a personal and social level.

## Author contributions

All authors listed have made a substantial, direct and intellectual contribution to the work, and approved it for publication.

### Conflict of interest statement

The authors declare that the research was conducted in the absence of any commercial or financial relationships that could be construed as a potential conflict of interest.
